# Comparative Sucrose Responsiveness in *Apis mellifera* and *A*. *cerana* Foragers

**DOI:** 10.1371/journal.pone.0079026

**Published:** 2013-10-23

**Authors:** Wenchao Yang, Haiou Kuang, Shanshan Wang, Jie Wang, Wei Liu, Zhenhong Wu, Yuanyuan Tian, Zachary Y. Huang, Xiaoqing Miao

**Affiliations:** 1 College of Honey Bee Science, Fujian Agriculture and Forestry University, Fuzhou, Fujian, China; 2 Research Institute of Eastern Honeybee, Yunnan Agriculture University, Kunming, Yunnan, China; 3 Department of Entomology, Michigan State University, East Lansing, Michigan, United States of America; University of Arizona, United States of America

## Abstract

In the European honey bee, *Apis mellifera*, pollen foragers have a higher sucrose responsiveness than nectar foragers when tested using a proboscis extension response (PER) assay. In addition, Africanized honey bees have a higher sucrose responsiveness than European honey bees. Based on the biology of the Eastern honey bee, *A. cerana*, we hypothesized that *A. cerana* should also have a higher responsiveness to sucrose than *A. mellifera*. To test this hypothesis, we compared the sucrose thresholds of pollen foragers and nectar foragers in both *A. cerana* and *A. mellifera* in Fujian Province, China. Pollen foragers were more responsive to sucrose than nectar foragers in both species, consistent with previous studies. However, contrary to our hypothesis, *A. mellifera* was more responsive than *A. cerana*. We also demonstrated that this higher sucrose responsiveness in *A. mellifera* was not due to differences in the colony environment by co-fostering two species of bees in the same mixed-species colonies. Because *A. mellifera* foragers were more responsive to sucrose, we predicted that their nectar foragers should bring in less concentrated nectar compared to that of *A. cerana*. However, we found no differences between the two species. We conclude that *A. cerana* shows a different pattern in sucrose responsiveness from that of Africanized bees. There may be other mechanisms that enable *A. cerana* to perform well in areas with sparse nectar resources.

## Introduction

The Asian hive bee, *Apis cerana* (Ac) is said to be better adapted to scattered nectar sources than the European honey bee, *A. mellifera* (Am) [1]. Both species are kept in China, with about 4 million colonies of Am and 3 million colonies of Ac [2]. Ac colonies are mainly kept in mountainous locations, while Am colonies are usually transported across the country to follow the blooming of flowers [3]. 

Proboscis extension response (PER) is the behavior of a honey bee responding by extending her proboscis when a drop of sucrose solution at sufficient concentration is applied to the antennae [4]. A PER assay can be used to test the responsiveness of honey bees to sucrose. The positive responses to a particular sugar concentration are totaled as a PER score. A higher PER score means a higher responsiveness to sucrose. This responsiveness is variable; it can be influenced by genotype, foraging experience, and the amount of food the bee has ingested [5,6]. 

Responsiveness to sucrose is associated with foraging choices. Bees with high PER scores (high responsiveness to sucrose) preferentially collect pollen and bees with low PER scores (low responsiveness to sucrose) mainly forage for nectar [7,8]. Sucrose responses in one-day-old bees can be used to predict foraging choice 2 to 3 weeks later. Young bees with the highest PER scores become water foragers, while bees with decreased PER scores become pollen foragers, nectar foragers, bees collecting both pollen and nectar, and “picky” foragers who return empty stomached [9]. 

The association between high PER scores and pollen-foraging is true across bee strains and/or subspecies. For example, an artificially selected high pollen-hoarding strain, which has a much higher proportion of pollen foragers (compared to unselected bees), also has a much higher PER score compared to the low pollen strain 7. In addition, a tropically evolved subspecies of the Western honey bee, Africanized honey bee (AHB, *Apis mellifera scutellata*) which invest more energy into brood rearing and swarming [10], also forage more for pollen, and have a higher PER score than Am [11,12]. PER scores at the emergence of Am workers can predict their learning abilities three weeks later [13]. PER is therefore a simple measurement that provides powerful insights into honey bee behavior and physiology.

Two lines of evidence suggest that Ac would have a higher PER score than Am. Because Ac is better adapted to mountainous areas and to areas with sparse nectar resources [1], we reasoned that they would be less picky about nectar resources (thus have a lower threshold/ higher PER score) as compared to Am. Scarcity would require them to use less desirable resources. In addition, Ac swarms more and produces less honey than Am, devoting more energy for reproduction [14,15]. These behaviors are more similar to that of AHB, suggesting that these two species would have similar PER scores. In this study we designed three experiments to understand the differences in sucrose responsiveness between and within Ac and Am foragers. In the first experiment we examine PER scores of pollen and nectar foragers of Ac and Am in their own colonies. In experiment 2, we examine PER scores of foragers of Ac and Am co-fostered in mixed-species colonies to remove the effect of different colony environment. Finally, we compared the sugar concentrations collected by nectar foragers of Ac and Am.

## Materials and Methods

### Honey bee colonies

Experiments and observations were conducted with colonies of *A. cerana* and *A. mellifera* from March to November, 2012 and April 2013 at apiaries of Fujian Agriculture and Forestry University, Fuzhou, Fujian, China. Colonies of Am (N = 15) were raised in front of the Bee Science Building (N 26°05’, E 119°13’), and 15 colonies Ac were at a park (N 26°05’, E 119°14’), 1 km east of the Bee Science Building.

### Experiment 1: %PER of pollen and nectar foragers from Ac and Am colonies

#### Experiment 1A

Honey bees for PER tests were collected from colony entrances of their own species. From each colony, 15 pollen foragers (with pollen loaded on corbiculae) and 15 nectar foragers (returning bees with no pollen) were sampled. Foragers were collected individually by coaxing them directly into a 1.5 ml Eppendorf tubes with a small hole drilled on the lid. These bees were then put on ice for ~3 min for immobilization. Each immobilized bee was harnessed in a drinking straw (5 mm diameter x 3 mm in height) with duct tape. Sugar solution was prepared with distilled water and sucrose (Sinopharm Chemical Reagent Co., Ltd, China) at concentrations of 0.1, 0.3, 1, 3, 10, and 30% (w/v). The harnessed bees were given at least 10 min for recovery before tests began. Distilled water was first used to test the honey bee’s response. If a bee responded to water that bee was fed water until she no longer responded to water. Then the 60 bees as a group (30 bees per species, with half of them pollen foragers) were tested sequentially with the series of sugar solution, starting with the lowest concentration. Water was used between each sugar solution to reduce possible sensory sensitization to antennal touch [6]. The inter-trial interval was 3 min. The PER was recorded as 1 if a bee extended her proboscis after both of her antennae were touched by a tooth prick pre-soaked with a sucrose solution, and 0 if she did not respond. We then expressed the responsiveness of 15 bees to each sucrose concentration as “percent of bees showing PER” (%PER) by dividing the number of bees that responded to sugar by the total number of tested bees. For example, if 5 bees out of 15 responded to 10% sugar solution, then the %PER for this concentration is 5/15= 33%. The %PER is a more refined response as compared to PER score, because the PER score is a summed response across all the sugar concentrations. We tested an equal number of colonies of Am and Ac on each day. A total of 10 colonies were tested for each species (with N=10 x 2 x 30=600 bees tested for both species).

#### Experiment 1B

Because Ac workers died at high proportions (>50%) either overnight, or even after only 1 hour of waiting, after being fed with 5μl 60% sugar solution, we used the above method to measure %PER in both species. However, the above method does not distinguish between hunger status (the reverse of satiation) and innate response, because pollen foragers might come home with an emptier stomach and respond more strongly to sucrose. Therefore we also tested workers in both species after a 5 min recovery, being fed 30μl 30% sugar solution, waiting for 90 min, and then determining their %PER. The other parts of methods were the same as the Experiment 1A. We measured 3 colonies of each species for this experiment (with N = 3 x 2 x 30 =180 bees tested for both species).

### Experiment 2: %PER of Ac and Am from mixed-species colonies

Three mixed-species colonies were set up, with two headed by Am queens and one by an Ac queen. Each colony started with two frames of sealed brood, one from each species. Newly emerged bees from both species were introduced into each colony and painted (N=200 per species per colony) to identify experimental bees from drifters from other colonies. When painted bees were 14 days old, 10 of these bees from each species were recovered from inside each colony and their %PER determined. At 21 days post colony setup, foragers of unknown ages (but at least 21 days old) of both species were captured and examined for their %PER (N = 3 x 2 x 20 = 120 bees). 

### Experiment 3: Sugar concentrations in nectar

To determine the sugar concentrations in the nectar brought in by foragers, 10 nectar foragers of each species were captured at hive entrances per hour, between 09:00 and 17:00 during May of 2012. The crop content of each bee was expressed with a pair of forceps and sugar concentrations were measured with a hand-held refractometer (0-90%, WZ-119/ATC). If the crop content of a worker had a sugar concentration < 2.5% she was considered a water forager and excluded from the data analysis. We tested one colony of each species per day with two observers, one for each species. The observers were then swapped for the bee species the next day to control for observer differences. For each species, about 7-10 bees were sampled per hour. We compared a total of 7 pairs of colonies over 7 days. We compared the average and the lowest sugar contents in nectar from bees collected during each hour for both species.

### Statistical analyses

For the PER assays, the dependent variable (%PER) was transformed with arcsine transformation (by taking the square root of %, then its arcsin) [16] and analyzed as a three-way ANOVA with sugar concentrations as repeated measures and forager type and bee species as the other two independent variables. All transformed data were checked for normality using the Komogorov-Smirnov Normality Test and all data sets were not significantly different from normal distributions (P > 0.05).

For nectar concentrations, the average and the lowest concentration during each hour for each bee species were compared using a paired t-test after arcsine transformation. Statview for Windows V5.01 (SAS Institute Inc., NC, USA) was used for all the tests.

## Results

### Experiment 1: %PER of pollen and nectar foragers from Ac and Am colonies

**Figure 1:**
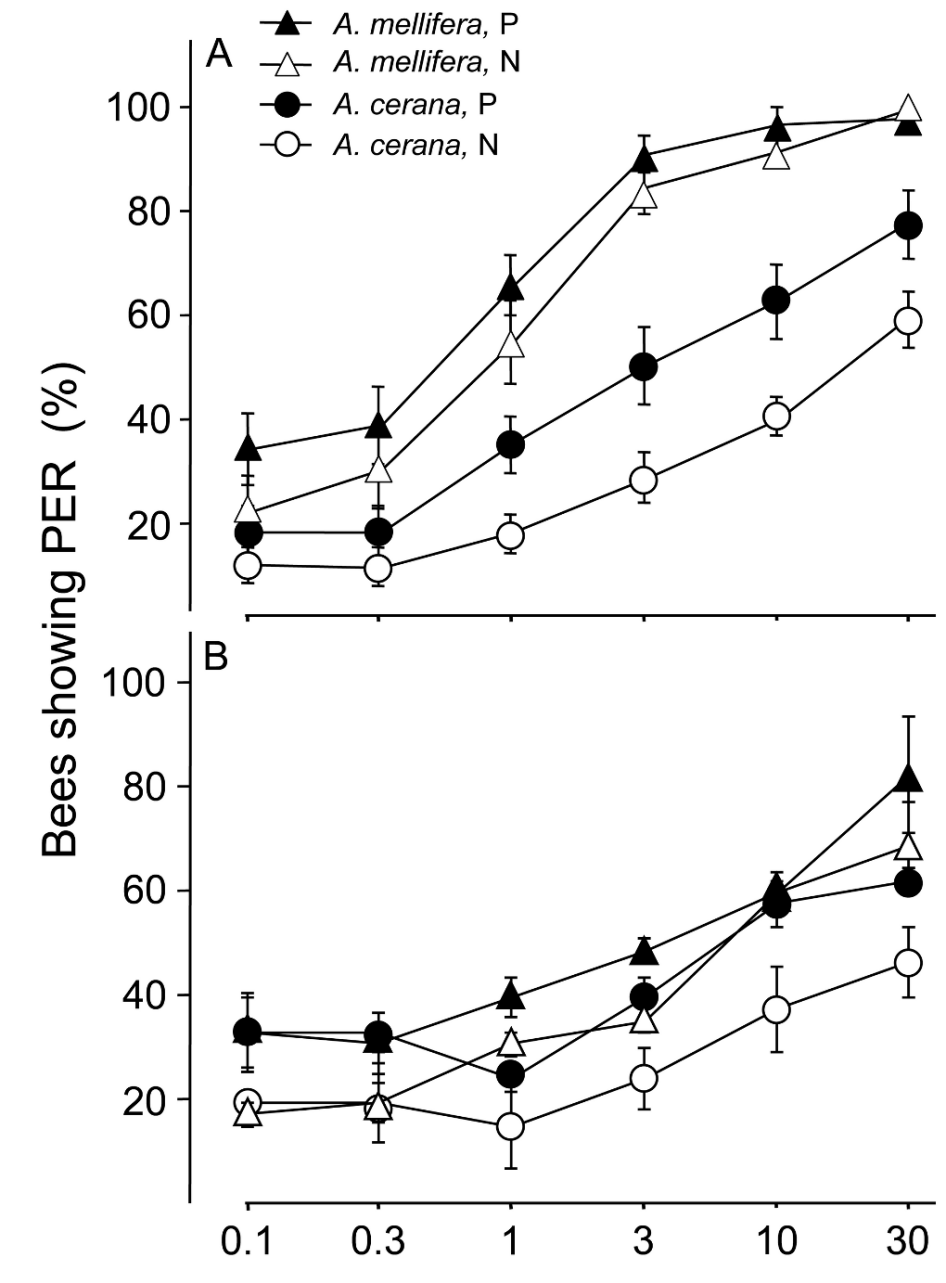
Percentage of bees showing proboscis extension response (mean+SE) of pollen (P, solid) and nectar (N, empty) foragers of *Apis mellifera* (triangles) and *A. cerana* (circles) in their own colonies, across different sucrose concentrations (plotted as log scale but with actual concentrations). A: unfed foragers tested directly, Data based on 10 colonies per species (N = 600 bees). B: foragers tested 30 min after being fed 5μl 30% sucrose solution. Data based on 5 colonies per species (N = 300 bees).

Experiment 1A: For unfed foragers from their own (species-pure) colonies, Am foragers were significantly more responsive than Ac foragers (F = 59.74, df = 1,36, P < 0.001). Across both species, pollen foragers were significantly more responsive than nectar foragers (F = 6.82, df = 1,36, P < 0.02, Figure 1A). There was no significant interaction between species and forager type (F=0.5, df = 1,36, P >0.4). In all groups, higher sugar concentrations always elicited higher %PER (F = 170.39, df = 5,180, P < 0.001). Except a significant interaction between bee species and sugar concentrations (F = 12.60, df = 5,180, P < 0.001), all other interactions with sugar concentrations were not significant (P > 0.05) between types of foragers and sugar concentrations and the three-way interactions (bee species x types of foragers x sucrose concentrations P > 0.05). 

Experiment 1B: For workers tested after being fed with 30 μl 30% sugar solution and recovering for 90 min, both types of Am foragers were significantly more responsive than Ac foragers (F = 6.69, df = 1,8, P < 0.03). Across the two species, pollen foragers were significantly more responsive than nectar foragers (F = 11.54, df = 1,8, P < 0.001, Figure 1B). In all groups, higher sugar concentrations always elicited higher %PER (F = 36.24, df = 5,40, P < 0.001). Interactions between bee species and type of foragers were not significant (F= 0.33, df = 1, 8, P > 0.5), neither was forager type x sugar concentration (F = 0.15, df = 5,40, P > 0.9), but interactions between species and sugar concentrations were significant (F = 3.48, df = 5,40, P < 0.02). There were no significant 3-way interactions (species x forage type x concentrations) (F = 0.53, df = 5,40, P >0.7). .16, df = 5,80, P < 0.05).

### Experiment 2: %PER of two species of bees from mixed-species colonies

**Figure 2:**
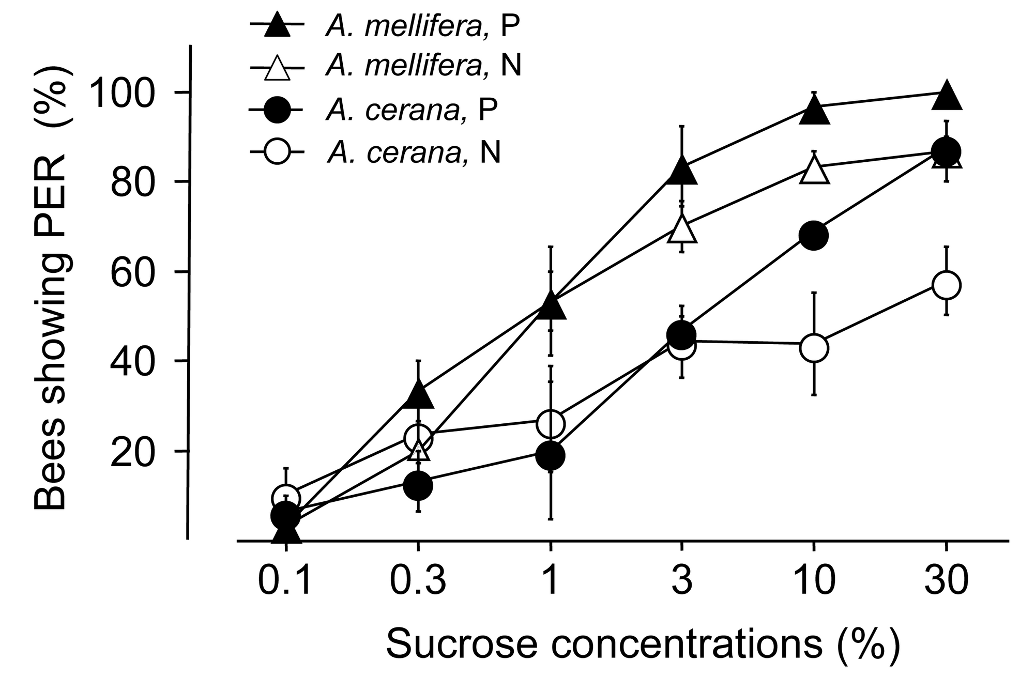
Percentage of bees showing proboscis extension response (mean+SE) of pollen (P, solid) and nectar (N, empty) foragers of *Apis mellifera* (triangles) and *A. cerana* (circles) of unknown-aged foragers in mixed-species colonies, across different sucrose concentrations (plotted as log scale but with actual concentrations). Data based on 3 mixed-species colonies (N = 3 x 2 x 2 x 10 = 120 bees).

**Figure 3:**
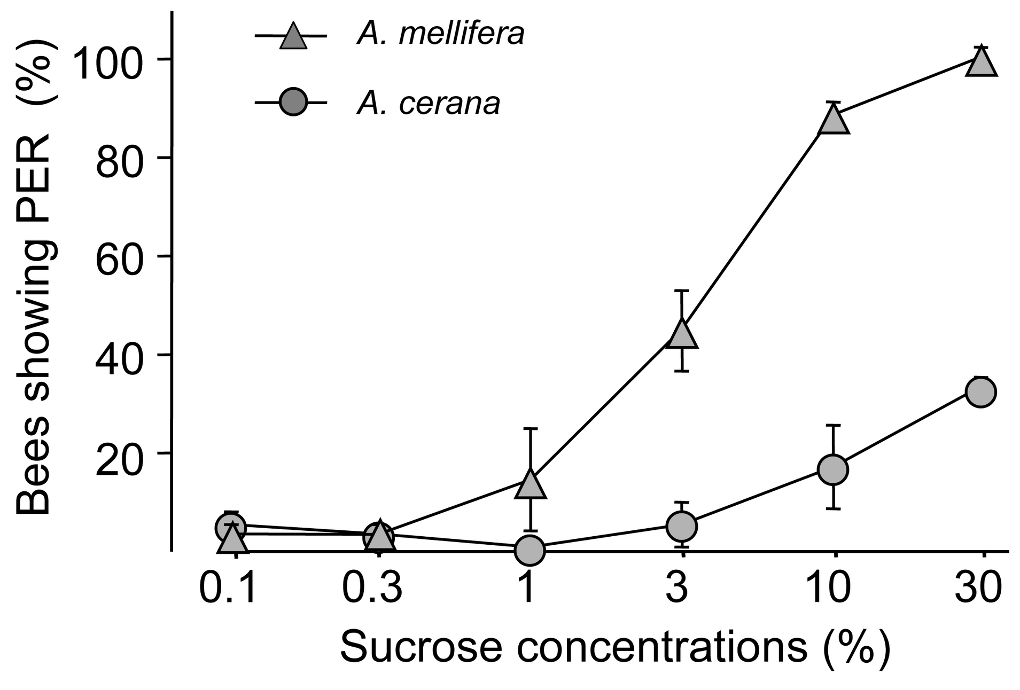
Percentage of bees showing proboscis extension response (mean+SE) of 14-day-old workers from three mixed-species colonies, of *Apis mellifera* (triangles) and *A. cerana* (circles). %PER were significantly higher in *A. mellifera* than *A. cerana*. Data based on 3 mixed-species colonies (N = 60 bees).

When raised within the same colony, Am foragers had a significantly higher %PER than Ac foragers, when foragers of all ages were examined (F = 14.88, df =1,8, P < 0.01, Figure 2). Pollen foragers were significantly more responsive than nectar foragers across both species (F = 7.23, df = 1,8, P < 0.05). The interactions between species and forager type were not significant (F = 0.92, df =1,8, P > 0.3). Again, concentrations of sugar significantly affected the %PER (F = 71.97, df = 5,40, P < 0.001). Interactions between types of foragers and sucrose concentrations were significant (F = 3.38, df = 5,40, P < 0.05), as were the interactions between species and sucrose concentrations (F = 5.64, df = 5,40, P < 0.01). Other interactions (bee species x types of foragers, and three-way interactions) were all found not significant (P > 0.05). The difference across the species also remained true in pre-foraging bees: the %PER for Am bees were significantly higher than Ac bees when they were tested at 14 days old (F = 18.89, df = 1,4, P < 0.02, Figure 3). Concentrations of sugar significantly affected the %PER (F = 40.42, df = 5,20, P < 0.001). Interactions between types of foragers and sucrose concentrations were significant (F = 11.33, df = 5,20, P < 0.001).

### Experiment 3: Sugar concentrations in nectar

**Figure 4:**
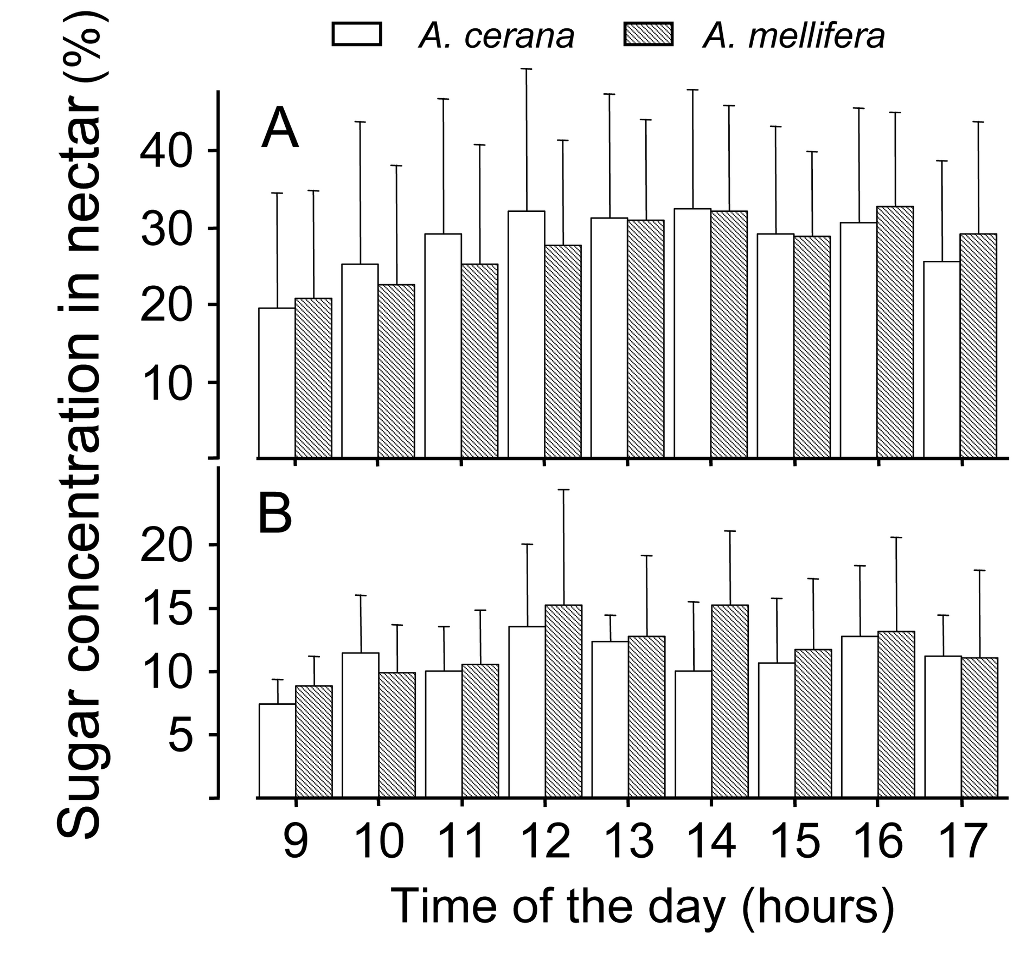
Sugar concentrations from crop of returning foragers of *A. cerana* (empty) and *A. mellfiera* (hatched). A: The average sugar concentrations of foragers caught each hour in both species; B: The lowest sugar concentration during each hour from foragers of both species.

The average sugar content of nectar collected each hour by Ac foragers was not significantly different from that collected by Am foragers (one tailed paired T-test, t = 1.00, P > 0.05, Figure 4a). The lowest sugar content of each hour (Figure 4b) was also not significantly different between the two species (one tailed paired T-test, t = 1.22, P > 0.05). ANOVA also failed to detect any differences between the two species using either variable (P > 0.05). The average hourly sugar concentrations in nectar varied significantly with time for both species (F = 17.97, df = 8,1102, P < 0.001), but the minimum hourly sugar concentrations did not vary with time (F = 1.35, df = 8,108, P > 0.05). 

## Discussion

This is the first study to report %PER in *Apis cerana*, and to demonstrate relative sucrose responsiveness between the two sister species, Ac and Am. We showed that 1). Pollen foragers in Ac have a higher sucrose responsiveness than nectar foragers, similar to what was previously reported in Am 2,8). Ac foragers show an overall lower sucrose responsiveness compared to Am; 3). The lower sucrose responsiveness in Ac is not due to their colony environment; co-fostered bees show the same species-specific difference; and 4). There are no significant differences between Ac and Am in sugar concentrations collected by nectar foragers. 

In the first half of Experiment 1 (Figure 1A), we did not feed honey bee workers and did a direct test, because when we used the standardized testing method, Ac experienced high mortality. To eliminate the possibility of confounding hunger status (because nectar foragers, by definition, will have more food storage than pollen foragers) and sucrose responsiveness, we also did a second test using bees that had been fed sugar (Figure 1B). The results were very similar. This suggests that in Experiment 1A, the differences in %PER were not due to hunger status alone but rather due to differences in sucrose responsiveness, or a combination of both factors.

In the first two experiments we used non-pollen foragers as a proxy for nectar foragers. In addition to nectar foragers, non-pollen foragers may also be empty-stomached foragers and water foragers. In a previous study, foragers with a crop content of less than 2.5% sugar were considered to be water foragers [17]. If we use this criterion for the foragers examined in our third experiment, nectar foragers comprised 91.3% of the total “non pollen foragers”. Because water foragers and empty-stomached foragers represent such a small percentage (<10%) of non-pollen foragers, we believe that non-pollen foragers can be used as a proxy for nectar foragers, and that the error due to this assumption should be negligible. Our usage of this classification is further supported by the fact that the relative responsiveness we found between forager types in Am in the first two experiments was in agreement with previous research, i.e. that pollen foragers show a higher responsiveness than nectar foragers.

Differences in foraging behavior in different races/subspecies of bees can be a result of differences in colony environments. A classic example is that AHB foragers, when fostered in a European bee colony, actually foraged later than European workers in the same colony, though they have a younger age of first foraging when raised in their own colony [18]. We eliminated the effect of the colony environment on forager responsiveness by creating mixed-species colonies using an established technique [19,20]. Workers from both species maintained their sucrose responsiveness difference even when they shared the same environment (Figure 2). They also maintained their sucrose responsiveness difference as middle aged (14-day-old) bees (Figure 3). These results indicate that our conclusion that Am has a higher sucrose responsiveness than Ac regardless of age or colony environment is robust. 

So why is it that Ac shows a very different sucrose responsiveness pattern from that of AHB when they have similar behavior patterns? AHB shows a higher responsiveness to sucrose than Am, and they also foraged less concentrated nectar, as predicted by their PER scores [21]. We hypothesized that Ac would likewise have a higher sucrose responsiveness than Am, because they forage in areas with more sparsely distributed nectar sources, therefore we infer that they should be less picky on nectar concentrations. But we found the reverse to be true in our species-pure colonies in Experiment 1 (Figure 1A, 1B). One possibility for this difference is that AHB is a tropically evolved honey bee while Ac and Am are both temperate bees, and somehow the differences between AHB and Am do not map the same way to Ac and Am. Because of their higher threshold for sucrose, we would expect that Ac bees would carry back nectar of a higher sugar concentration than Am, but when we compared the concentrations on the same days across the same hours between the two species, this was not the case. It is not clear why Ac, once again, failed to fit a predicted pattern. 

 Although during May (2012) in Fuzhou, Fujian Province the nectar resources were rather poor as was usual during this time of year, *A. mellifera* colonies did not need to be fed sugar to maintain their colonies. Because there were no major cultivated crops/trees blooming in June, available nectar resources were more similar to mountainous areas during that time. However, it would be interesting to compare sugar concentrations between the two species during a major honey flow as well as during a major nectar dearth when Am colonies need to be fed. 

Other mechanisms instead of lowered sucrose threshold may contribute to the ability of Ac to survive better than Am under poor food conditions. Foragers of Ac leave their colonies earlier in the morning and end their foraging later during the evening [22]. Foragers of Ac may make more foraging trips per day than foragers of Am (no data yet). Ac is known to perform significantly better in learning on both color and grating patterns than Am [23], so perhaps they can locate sparse nectar resources more easily. Another difference may be metabolic rates. Oxygen consumption rates in Am worker pupae and adults are higher than that of Ac [24,25]. Am colonies therefore have to consume more sugar to maintain higher metabolic rates. Further research on differences in foraging trips, food consumption, the age of first foraging, or the percentage of bees foraging in the two species will help explain why Ac is better adapted to scattered nectar sources. 
