# Correction: Comparative Sucrose Responsiveness in *Apis mellifera* and *A*. *cerana* Foragers

**DOI:** 10.1371/annotation/20dfdf0f-eab5-4ed6-ba0f-37b55b5ccf6a

**Published:** 2013-12-11

**Authors:** Wenchao Yang, Haiou Kuang, Shanshan Wang, Jie Wang, Wei Liu, Zhenhong Wu, Yuanyuan Tian, Zachary Y. Huang, Xiaoqing Miao

There was an error in the legend of Figure 1B, "Percentage of bees showing proboscis extension response (mean+SE) of pollen (P, solid) and nectar (N, empty) foragers of Apis mellifera (triangles) and A. cerana (circles) in their own colonies, across different sucrose concentrations (plotted as log scale but with actual concentrations)." The correct Figure 1B legend is:

B: foragers tested 90 min after being fed 30μl 30% sucrose solution. Data based on 3 colonies per species (N = 90 bees per species). 

